# Identification and Characterization of DcUSAGT1, a UDP-Glucose: Sinapic Acid Glucosyltransferase from Purple Carrot Taproots

**DOI:** 10.1371/journal.pone.0154938

**Published:** 2016-05-12

**Authors:** Yi-Yun Chen, Zhi-Sheng Xu, Ai-Sheng Xiong

**Affiliations:** State Key Laboratory of Crop Genetics and Germplasm Enhancement, College of Horticulture, Nanjing Agricultural University, 1 Weigang, Nanjing, China; Saint Mary's University, CANADA

## Abstract

Purple carrots accumulate abundant cyanidin-based anthocyanins in taproots. UDP-glucose: sinapic acid glucosyltransferase (USAGT) can transfer the glucose moiety to the carboxyl group of sinapic acid thereby forming the ester bond between the carboxyl-C and the C1 of glucose (1-*O*-sinapoylglucose). 1-*O*-sinapoylglucose can serve as an acyl donor in acylation of anthocyanins and generate cyanidin 3-xylosyl (sinapoylglucosyl) galactoside in purple carrots. This final product helps stabilize the accumulation of anthocyanins. In this study, a gene named *DcUSAGT1* encoding USAGT was cloned from ‘Deep purple’ carrot taproots. Enzymatic activity was determined using high performance liquid chromatography (HPLC). The optimal temperature and pH value were 30°C and 7.0, respectively. Kinetic analysis suggested a *K*_m_ (sinapic acid) of 0.59 mM. Expression profiles of *DcUSAGT1* showed high expression levels in the taproots of all the three purple carrot cultivars but low expression levels in those of non-purple carrot cultivars. The USAGT activity of different carrots *in vitro* indicated that crude enzyme extracted from the purple carrot taproots rather than non-purple carrot taproots exhibited USAGT activity. These results indicated that DcUSAGT1 may influence anthocyanin biosynthesis of purple carrot taproots.

## Introduction

As described in detail previously, carrots (*Daucus carota* L.) located in the second place in the aspect of economic value among all the vegetables in the European Union. They are also important vegetable crops worldwide [1, 2]. Carrots are biannual diploids (2n = 2x = 18) with a comparatively small genome about 470 Mb [3]. In the large family of Apiaceae, carrot is the most typical species at the genetic and molecular levels [4, 5]. Carrots are rich in a variety of nutrition, such as carotenoids, hydroxycinnamic acids, anthocyanins, potassium, vitamin B6, vitamin C, folic acid, and magnesium [6]. Purple carrots (*D*. *carota* ssp. *sativus* var. *atrorubens* Alef.) particularly accumulate rich anthocyanins in taproots. Anthocyanins are antioxidants that can enhance memory and vision [7]. Under natural conditions, free anthocyanins are rare and are usually acylated with sinapic, ferulic, and coumaric acids in the last step of anthocyanin biosynthesis [8].

**Fig 1:**
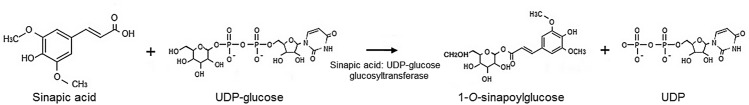
1-*O*-sinapoylglucose biosynthesis pathway. Sinapic acid and UDP-glucose act as potential substrates for the UDP-glucose: sinapic acid glucosyltransferase, which catalyzes the transfer of glucose moiety from UDP-glucose to the 1-*O*-position of sinapic acid.

The involvement of UDP-glucose is well identified in the formation of phenylpropanoid glycosides. Sinapic acid is a major phenylpropanoid participating in two different pathways, which operates at different stages and in different tissues during the development of plants [9]. Glucosyltransferases play important roles in the formation of the intermediates during these pathways. One pathway produces sinapoylmalate and sinapoylcholine through the synthesis of 1-*O*-sinapoylglucose [10]. The other pathway goes through the offspring of sinapyl alcohol-4-*O*-glucoside, generating syringyl units found in lignin [11]. UDP-glucose: sinapic acid glucosyltransferase (USAGT) participates in the first pathway (Fig 1). It is an essential enzyme during the transfer of sinapic acid to the product of 1-*O*-sinapoyl-glucose.

UDP-glucose glucosyltransferase activity has been detected in many plants, such as UDP-glucose: flavonol 3-*O*-glucosyltransferase from soybean cell cultures [12], cyanidin 3-*O*-glucosyltransferase from flower cell cultures [13], UDP-glucose: thiohydroximate glucosyltransferase from seedlings of *Brassica napus* [14], and USAGT from cultured cell extracts of *D*. *carota* [15]. Although the enzymatic activity of these glucosyltransferases has been recognized for many years, most of their corresponding genes have not been identified [16]. The gene encoding for USAGT was cloned from *B*. *napus* [17].

However, the USAGT-gene in carrot has not been identified in detail. Furthermore, the potential function of USAGT in anthocyanin biosynthesis of purple carrot taproots has not been studied yet. Our work aimed to investigate the role of USAGT in anthocyanin biosynthesis of purple carrot taproots. Here, a gene named *DcUSAGT1* that encodes for USAGT was identified from purple carrot taproots. We also reported the purification of rDcUSAGT1 (recombinant DcUSAGT1) protein and detected rDcUSAGT1 activity.

DcUSAGT1 may play important roles in the stability of anthocyanin accumulation, the expression levels of *DcUSAGT1* gene in the purple carrot taproots may be higher than those in non-purple carrot taproots. Three purple carrot cultivars ‘Deep purple’, ‘Purple68’, ‘Tianzi2hao’, and three non-purple carrot cultivars ‘Kurodagosun’, ‘Sanhongliucun’, ‘Junchuanhong’ were selected for expression profiles performance to verify this assumption. Expression patterns of *DcUSAGT1*and crude enzyme of DcUSAGT1 in purple and non-purple carrots were also detected and analyzed. Those results will help us to further determine the effect of DcUSAGT1 on cyanidin-based anthocyanin biosynthesis.

## Materials and Methods

### Sequence database construction

The template sequence for database search is glycosyltransferase (AT3G21560) from *Arabidopsis thaliana*. Then the sequences of *DcUSAGT1* gene were retrieved from CarrotDB, a genomic and transcriptomic database for carrots built by our group (http://apiaceae.njau.edu.cn/carrotdb/) [18]. The nucleotide sequence was submitted to GenBank and assigned a GenBank accession number of KT595241. All primers used in this study are listed in Table 1.

**Table 1:** Primer sequences of *DcUSAGT1* gene and the primer sequences used for Quantitative Real time PCR amplification of *Dcactin* and *DcUSAGT1* gene.

Name	Oligonucleotides sequences
DcUSAGT1-Forward primer	ATGAGCCCTGTATATCAAGACTCCA
DcUSAGT1-Reverse primer	TCAGTTCATCATCAGCTTATCCACA
Dcactin-Forward primer	CGGTATTGTGTTGGACTCTGGTGAT
Dcactin-Reverse primer	CAGCAAGGTCAAGACGGAGTATGG
DcUSAGT1-QForward primer	TTCGTAATGCAGTGCTTTGGGT
DcUSAGT1-QReverse primer	GCTGGATAAGGAGTTGAAGGGTGT

### Structure prediction and structure-based sequence alignment

Protein 3D structures were predicted on the Phyre2 website (http://www.sbg.bio.ic.ac.uk/phyre2/html/page.cgi?id=index) [19]. The domain of the proteins was analyzed using the Conserved Domain Database of the NCBI website. DNAMAN 6.0 software was used to complete the multiple sequence alignments of UDP-glucose: glucosyltransferase domains.

### Plant material and treatment

This experiment was conducted in the State Key Laboratory of Crop Genetics and Germplasm Enhancement of Nanjing Agricultural University. The carrot seeds: purple carrot cultivars ‘Deep purple’, ‘Purple68’, and ‘Tianzi2hao’, non-purple carrot cultivars ‘Kurodagosun’, ‘Sanhongliucun’, and ‘Junchuanhong’ used in this study were kept in our Lab (Lab of Apiaceae Plant Genetics and Germplasm Enhancement, Nanjing Agricultural University). After seven-day germination, carrots were transferred into plates which mixed with organic soil and vermiculite (3:1). The artificial climate chamber programmed for 12 h/12 h day/night at 25°C/18°C with a relative humidity of 60%–70%. The intensity of illumination is 300 μmol∙m^-2^s^-1^.

For gene cloning, the whole taproots of purple carrot cultivar ‘Deep purple’ were harvested after two months. For expression profiles experiment, whole taproots of three purple carrot cultivars and three non-purple carrot cultivars were harvested after two, three, and four months, respectively. For the USAGT activity test *in vitro*, two-month taproots of two carrot cultivars ‘Deep purple’ and ‘Kurodagosun’ were harvested. All samples were immediately frozen in liquid nitrogen and stored at –80°C in a refrigerator before RNA extraction. The chemical reagents used for HPLC analysis in this study, such as sinapic acid and UDP-glucose, were purchased from Sigma (San Francisco, USA).

### Constructing the expression vector to host the *DcUSAGT1* gene

Total RNA from the taproot tissues of carrot cultivar ‘Deep purple’ were extracted using a total RNA kit (RNA Simply, Tiangen, Beijing, China) according to the manufacturer’s protocol. Reverse transcription into cDNA was conducted using PrimeScript RT reagent kit (TaKaRa, Dalian, China). cDNA was synthesized using 5 μg of total RNA as the template of the reverse transcription system in a 20 μL reaction volume. The *DcUSAGT1* gene was amplified using diluted cDNA as the template with *DcUSAGT1*-Forward primer and *DcUSAGT1*-Reverse primer, respectively (Table 1). The *DcUSAGT1* gene was cloned by polymerase chain reaction (PCR) using a forward primer 5'-ATGAGCCCTGTATATCAAGACTCCA-3' and a reverse primer 5'-TCAGTTCATCATCAGCTTATCCACA-3', and was then sequenced by Applied Biosystems 3730 DNA Analyzer (Genscript, Nanjing, China). The PCR conditions were as follows: 95°C for 5 min; followed by 35 cycles of 94°C for 30 s, 54°C for 15 s, and 72°C for 60 s; and 72°C for 10 min. The PCR product was recovered and inserted in a plasmid pMD-19 vector (TaKaRa, Dalian, China), and the ligation mixture was applied to transform the *Escherichia coli* strain (DH5α). The ampicillin resistant bacteria in the LB medium were identified, and plasmid DNA was sequenced afterward (Genscript, Nanjing, China). The *DcUSAGT1* gene was then inserted into a pET-30a (+) expression vector (Novagen, Darmstadt, Germany) with the *Sac* Ӏ/*Hin*d ӀӀӀ sites and transformed into *E*. *coli* DH5α. The authenticity of the result was confirmed by PCR analysis and sequencing.

### Purification of DcUSAGT1 protein

To produce DcUSAGT1 protein, a single colony of *E*. *coli* BL21DE3 cells (TransGen, Beijing, China) carrying recombinant plasmid pET-30a (+)-DcUSAGT1 was grown in 50 mL of fresh LB medium containing 50 mg/mL kanamyacin until OD_600_ value reached 0.4 to 0.6 at 37°C. The recombinant protein was then induced with the addition of 1 mM IPTG at 18°C, whereas the control culture lacked IPTG induction. After over 12 h of induction, the cells were harvested by centrifugation for 8 min (4°C, 4,000 × *g*) and re-suspended in 2 mL lysis buffer (pH = 7.5) containing 50 mM NaH_2_PO_4_, 300 mM NaCl, 10% glycerol, 10 mM β-mercaptoethanol, and 10 mM imidazole. The suspension was then sonicated on ice for 20 min. The mixture was centrifuged for 10 min (4°C, 12,000 × g) and filtered with 0.22 μm microfiltration membranes.

The protein was purified in a column containing Ni-NTA-agarose resin (1.5 mL bed volume) according to the instructions from the QIA-expression system (Qiagen, Hilden, Germany). The recombinant protein fraction was desalted in buffer (pH 7.5, 50 mM NaH_2_PO_4_-Na_2_HPO_4_, 0.1 mM EDTA, and 1 mM DTT) using HiTrap Desalting Column (GE, Fairfield, USA) according to the manufacturer’s instructions. The purified protein assay was quantified using Bradford assay kit with BSA as standard.

### Enzyme activity assay

The assay mix (100 μL) was consisted of 5 μL recombinant protein, 5 mM sinapic acid, 50 mM UDP-glucose. The activity was performed in 50 mM buffer (NaH_2_PO_4_-Na_2_HPO_4_, pH 7.0) at 30°C for 30 min. The enzyme which was boiled at 99°C for 5 min was taken as control. After incubation, the reaction was terminated by adding 100 μL absolute ethyl alcohol. A total of 20 μL of the supernatant was used for analysis by HPLC.

### Optimum pH and temperature of the enzyme activity

The determination of optimum pH for enzyme activity was performed in 50 mM NaH_2_PO_4_-Na_2_HPO_4_ at different pH values from 6.0–8.0 and in 50 mM glycine-NaOH (pH 8.5–9.5) at 35°C for 3 h. The optimum temperature for enzyme activity was determined at pH 7.0 in 50 mM NaH_2_PO_4_-Na_2_HPO_4_ at different temperatures (20–50°C) for 3 h.

### Optimal kinetic analysis of the enzyme activity

The reactions contained fixed UDP-glucose concentration of 50 mM and sinapic acid concentration varying from 0.05 mM to 1.6 mM in 50 mM NaH_2_PO_4_-Na_2_HPO_4_. The reaction products were carried out in pH 7.0 at 30°C for 3 h according to the pH optima and linearity of the two preceding activities. Afterward, the reaction was stopped by the addition of 100 μL of quick-frozen ethanol and stored at –20°C before the HPLC analysis.

### HPLC analysis

Enzymatic activity was determined by HPLC in absorbance at 306 nm. Precision instrument (Agilent Technologies1200 series) with a Columbus C18 column (4.6×150 mm) was used for HPLC analysis. Acetonitrile in H_2_O (containing 1% glacial acetic acid) was used as mobile phase. Separations were accomplished for 20 min in a linear gradient from 10% to 40% acetonitrile in H_2_O with the flow rate of 1 mL/min.

### Expression profile analysis of *DcUSAGT1* gene in purple and non-purple carrot cultivars

Quantitative real-time polymerase chain reaction (qRT-PCR) systems were performed on ABI7500 (Applied Biosystems,USA) with SYBR Premix *Ex Taq* (TaKaRa, Dalian, China). Each reaction was repeated three times with independent RNA samples for relative accuracy. Expression levels of mRNA for *DcUSAGT1* gene were measured using the primer pairs of *Dcactin* gene for qRT-PCR expression analysis [20]. All primer pairs used in qRT-PCR reaction are listed in Table 1. Reaction volume (20 μL) was composed of 10 μL of SYBR Premix *Ex Taq*, 7.2 μL of ddH_2_O, 0.4 μL each of primer, and 2 μL of diluted cDNA. qRT-PCR conditions were 95°C for 30 s, followed by 40 cycles of 95°C for 10 s and 60°C for 30 s, and finally 65°C for 15 s.

### Determination of the USAGT activity of carrot in *vitro*

The purple carrot ‘Deep purple’ and non-purple carrot ‘Kurodagosun’ were selected to extract crude enzyme for DcUSAGT1 activity determination. The reaction system was the same to the glucosyltransferase activity assay described previously. A total of 20 μL of the supernatant was used for analysis by HPLC.

## Results

### Sequence and structure analysis of DcUSAGT1

**Fig 2:**
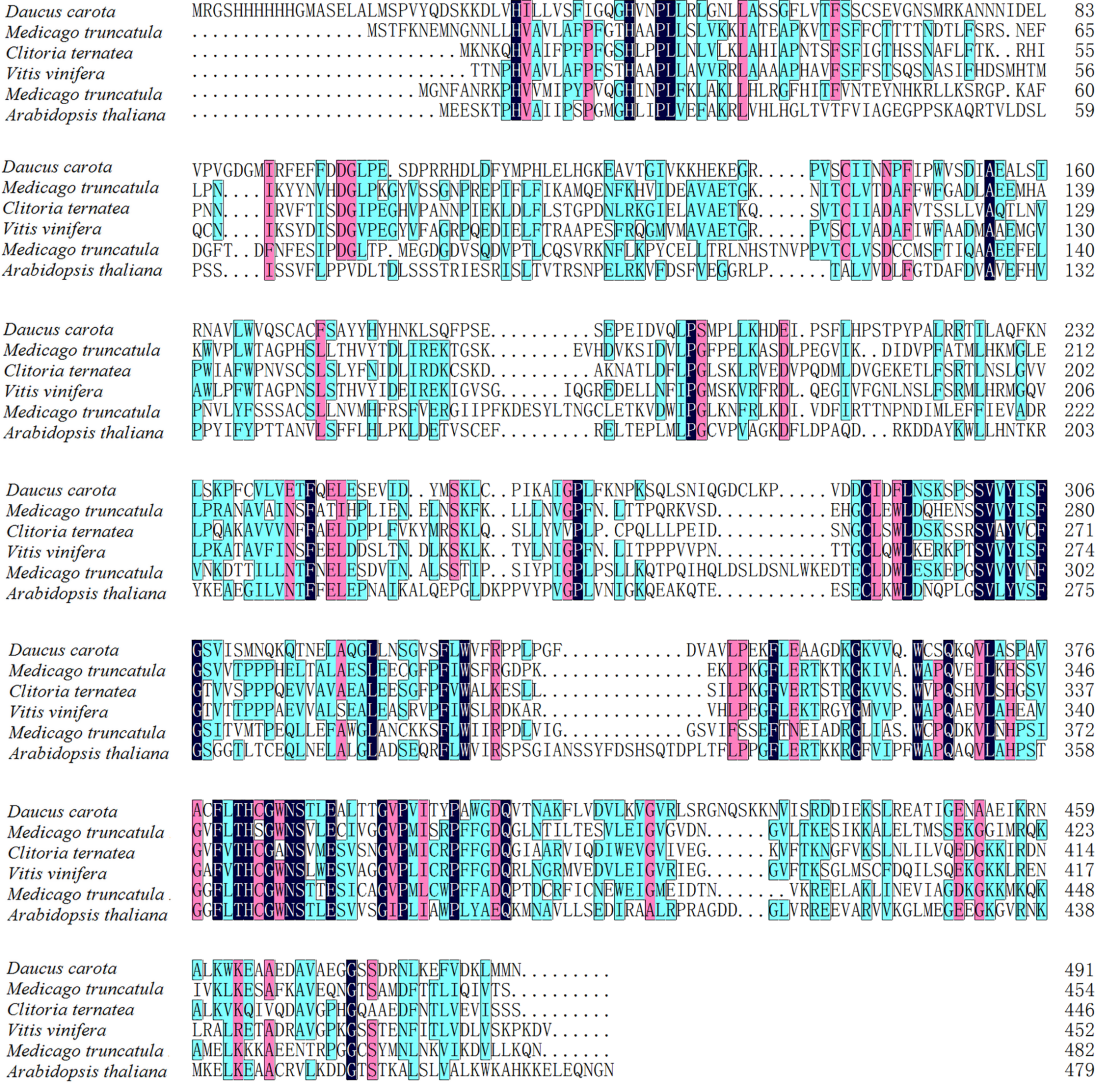
Structure-based domain sequence alignments of DcUSAGT1 with UDP-glucose: glucosyltransferases in other plants.

The result of cloning of the cDNA showed that the gene *DcUSAGT1* has an open reading frame of 1,473 bp encoding 491 amino acids. The calculated relative molecular mass (Mr) of DcUSAGT1 is 54.55 kDa. Based on NCBI search, the protein contains conserved domains of the glycosyltransferase family, which belongs to the glycosyltransferase-B type superfamily. Structure-based sequence alignment was performed on the DcUSAGT1 of other plant UDP-glucose: glucosyltransferases (Fig 2), such as UDP-glucose: flavonoid 3-*O*-glucosyltransferase from *Medicago truncatula* (Accession No. XP_003610163.1), UDP-glucose: anthocyanidin 3-*O*-glucosyltransferase from *Clitoria ternatea* (Accession No. 3WC4_A), UDP-glucose: flavonoid 3-*O*-glucosyltransferase from *Vitis vinifera* (Accession No. AAB81682.1), cytokinin-*O*-glucosyltransferase from *M*. *truncatula* (Accession No. XP_003618665.1), and UDP-glycosyltransferase 72B1 (UGT72B1) from *A*. *thaliana* (Accession No. NP_192016.1). The consensus of domain sequence was 42.19%.

**Fig 3:**
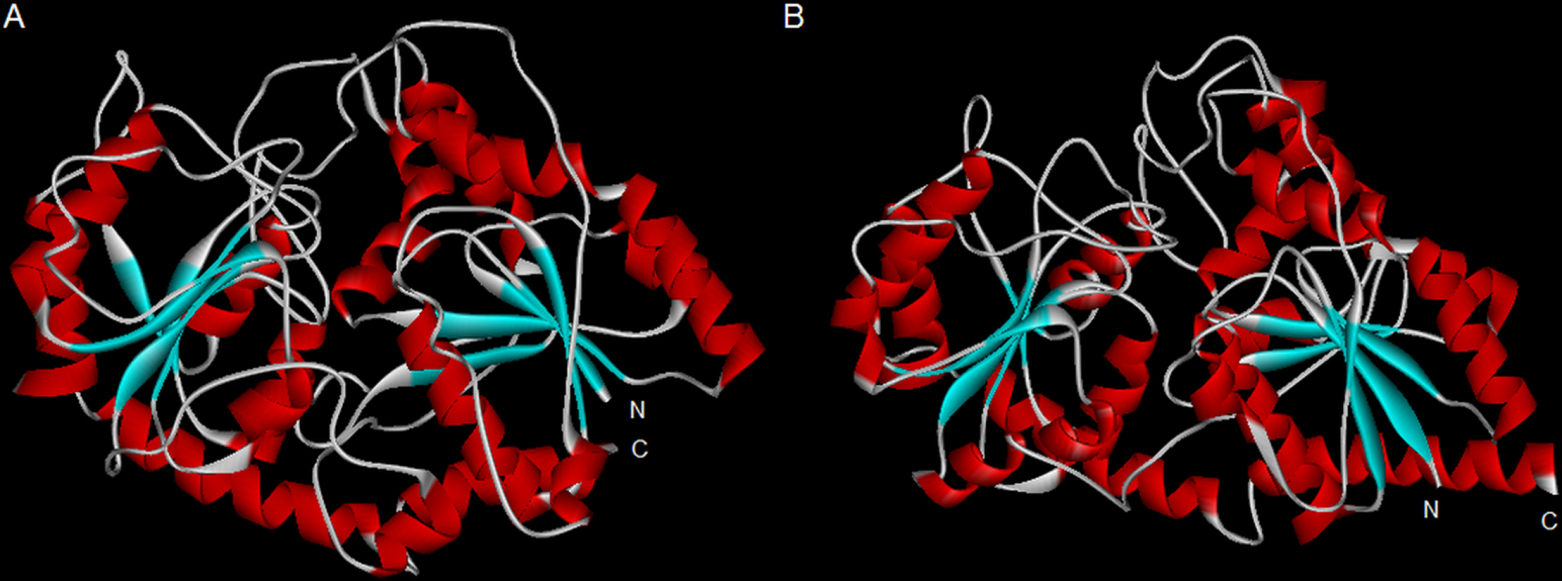
Predicted 3D structure of UGT72B1 and DcUSAGT1. (A) 3D structure of UGT72B1. (B) 3D structure of DcUSAGT1. N represents the N-terminal, C represents the C-terminal. Red-helix represents α-helix, blue-sheet represents β-sheet. The long deep cleft in the middle of the structure is used for substrate binding.

3D structure was constructed based on UGT72B1 as the closest model. DcUSAGT1 displays 25% identity with UGT72B1. DcUSAGT1 and UGT72B1 both belong to glycosyltransferase-B type superfamily, as expected of the glycosyltransferase-1 enzyme family. The glycosyltransferase-B structure exists in the domain of DcUSAGT1 and UGT72B1. DcUSAGT1 features of N- and C-terminal domains, which is similar to those of UGT72B1. In DcUSAGT1, the N-terminal domain contains eight alpha helixes and five beta sheets, whereas the C-terminal covers seven alpha helixes and six beta sheets. However, eight alpha helixes and six beta sheets were observed in the N-terminal of UGT72B1, whereas nine alpha helixes and six beta sheets occurred in the C-terminal. These two domains were separated by a deep cleft for substrate binding (Fig 3).

### Screening for rDcUSAGT1 activity

**Fig 4:**
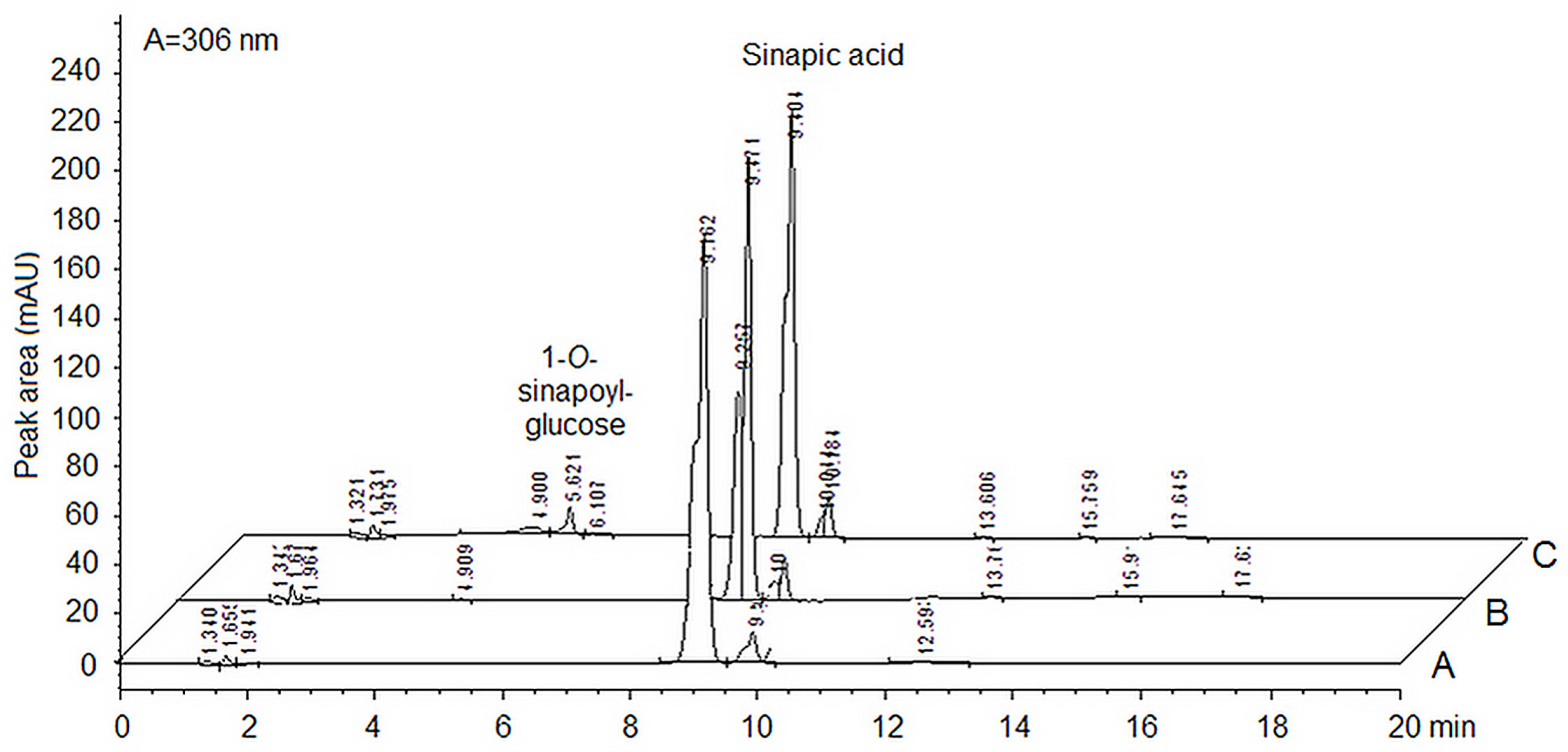
Chromatograms of product formation by the purified enzyme of UDP-glucose: sinapic acid glucosyltransferase in carrots by HPLC. Peak area A represents the reaction mix without enzyme as the blank control. Peak area B represents the reaction volume with deactivated enzyme. Peak area C represents the reaction system with active enzyme. The retention time of the product: 1-*O*-sinapoyl-glucose was about 5.6 min, and the peaks for sinapic acid were 9.1 min in average.

Initial screening for rDcUSAGT1 activity was carried out at pH 7.0 (50 mM NaH_2_PO_4_-Na_2_HPO_4_) under 30°C for 30 min. The retention time of product (1-*O*-sinapoylglucose) and substrate (sinapic acid) was about 5.6 and 9.1 min, respectively (Fig 4). Comparing the control (using deactivated rDcUSAGT1) and the blank control, no product activity was detected.

### Effect of temperature and optimum pH

**Fig 5:**
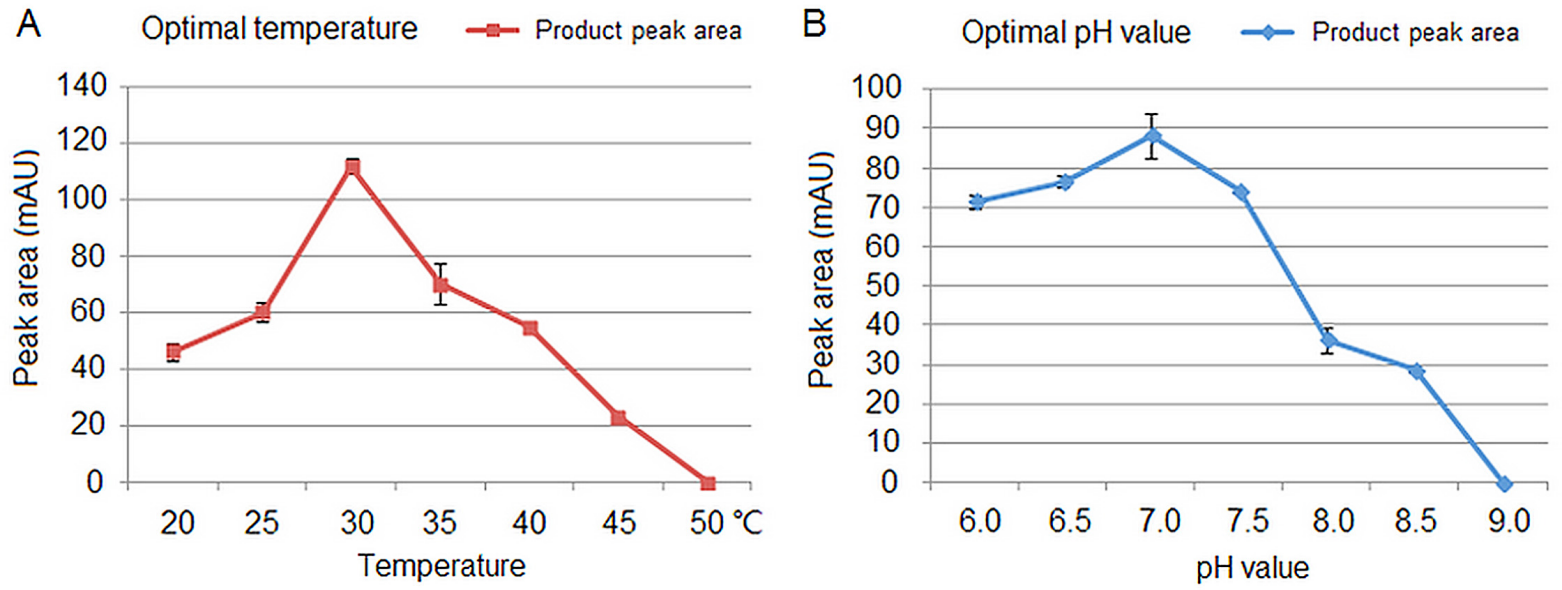
Effects of temperature and optimal pH value of rDcUSAGT1 activity with sinapic acid as substrate. (A) Effect of temperature. (B) Optimal pH value. The values are the means of three separate experiments with error bars showing ± SD.

rDcUSAGT1 activity was determined at temperatures from 20°C to 50°C and the optimal temperature for rDcUSAGT1 activity was 30°C (Fig 5A). Whether the temperature increased or decreased, the activities always gradually declined. When the temperature reached 50°C, rDcUSAGT1 activity was not detectable. The optimum pH for the reaction was 7.0 in a pH range of 6.5–7.5 (Fig 5B), and the activity of rDcUSAGT1 stopped when the pH exceeded 9.0.

### Kinetic parameters of rDcUSAGT1

The pattern of dependence of the rDcUSAGT1 reaction rate was consistent with Michaelis-Menten kinetic behavior. *K*_m_ and *V*_max_ values were estimated based on a Hanes-Woolf plot. *K*_cat_ value was calculated using the derived rDcUSAGT1 Mr of 54,556 g.mol^-1^. For the purified enzyme, the calculated *V*_max_, *K*_m_, *K*_cat_, and *K*_cat_/*K*_m_ values of sinapic acid as substrate are listed in Table 2. The calculated *V*_max_ was 0.74 nk_cat_ mg^-1^, *K*_m_ was 0.59 mM, *K*_cat_ was 0.04 s^-1^, and *K*_cat_/*K*_m_ was 67.79 M^-1^s^-1^.

**Table 2:** Kinetic analysis data of rDcUSAGT1.

Substrate	*V*_max_ (nk_cat_ mg^-1^)	*K*_m_ (mM)	*K*_cat_ (s^-1^)	*K*_cat_/*K*_m_ (M^-1^s^-1^)
Sinapic acid	0.74	0.59	0.04	67.79

### Expression profiles of *DcUSAGT1* in the taproots of six carrot cultivars at different stages

**Fig 6:**
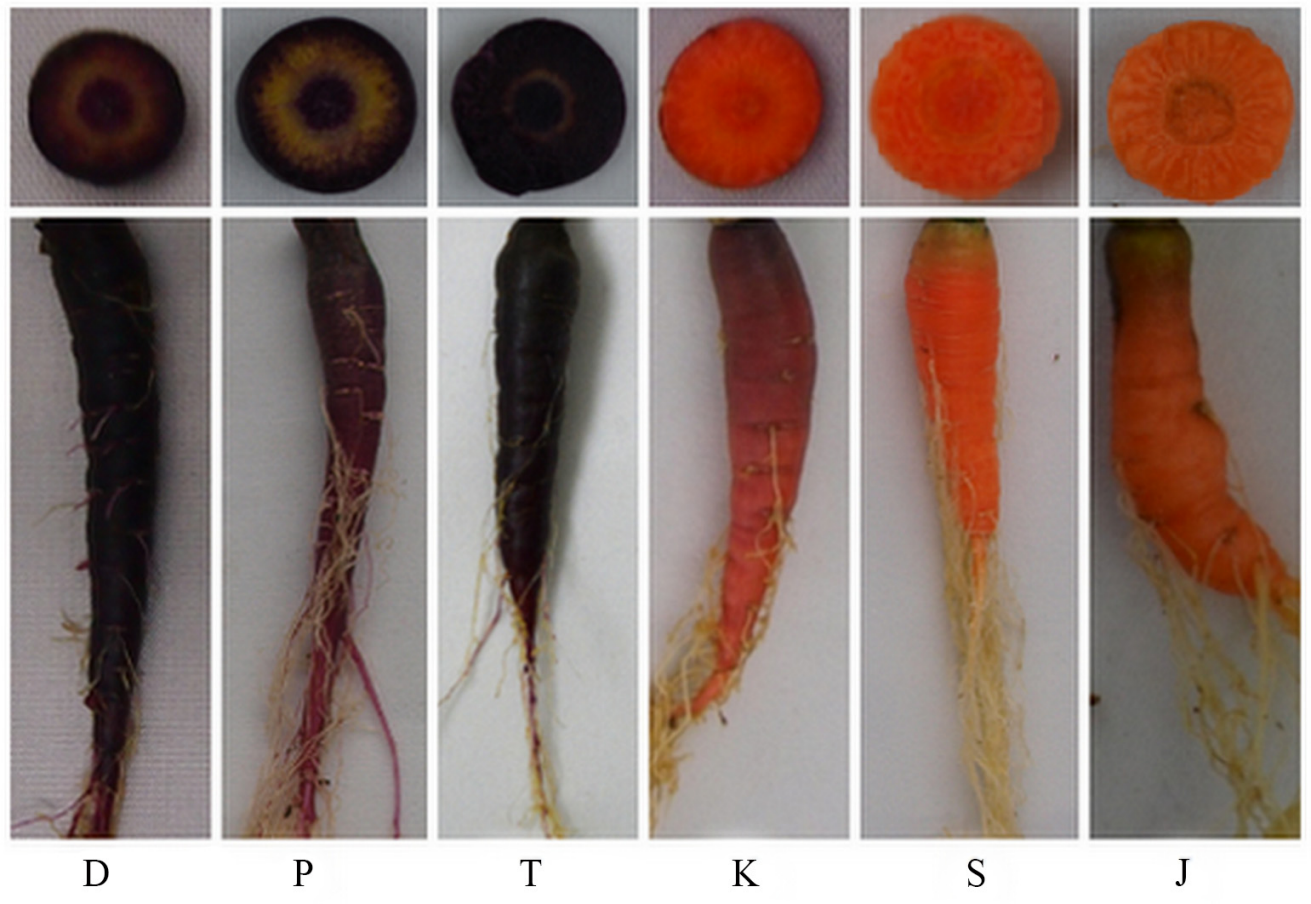
Photos of purple and non-purple carrot cultivars used in this study. D represents ‘Deep purple’, T represents ‘Tianzi2hao’, and P represents ‘purple68’; S represents ‘Sanhongliucun’, K represents ‘Kurodagosun’, and J represents ‘Junchuanhong’.

**Fig 7:**
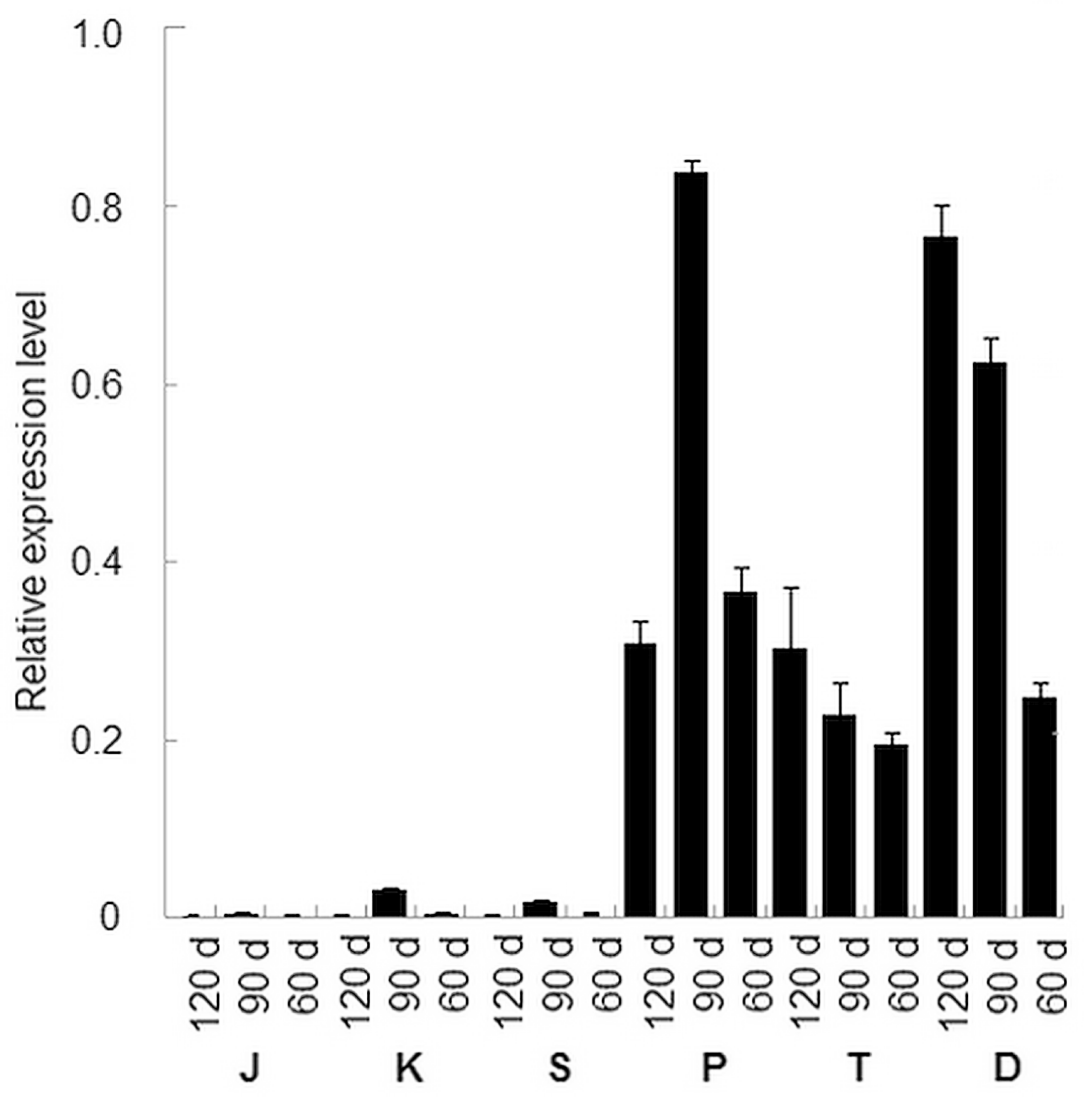
Expression profiles of *DcUSAGT1* gene in three purple carrot cultivars and three other non-purple carrot cultivars. The mRNA level of *Dcactin* was defined as 1. The values are the means from triplicate qRT-PCR analysis with error bars showing ± SD. D represents ‘Deep purple’, T represents ‘Tianzi2hao’, and P represents ‘purple68’; S represents ‘Sanhongliucun’, K represents ‘Kurodagosun’, and J represents ‘Junchuanhong’.

A total of 18 samples were chosen to perform qRT-PCR analysis (Fig 6). The expression profiles indicated that the *DcUSAGT1* gene was up-regulated more significantly in purple carrots (‘Deep purple’, ‘Purple68’, and ‘Tianzi2hao’) than in non-purple carrots (‘Kurodagosun’, ‘Sanhongliucun’, and ‘Junchuanhong’) (Fig 7). The trend of gene expression levels in the root development stage of the non-purple carrots (90 d > 60 d > 120 d) was similar to that of the purple carrot ‘Purple68’. The trend of *DcUSAGT1* expression levels in the taproots of ‘Deep purple’ and ‘Tianzi2hao’ cultivars was 120 d > 90 d > 60 d. Among the purple carrots, the expression level of *DcUSAGT1* gene from purple carrot was highest in ‘Purple68’ (at the stage of 90 d), followed by ‘Deep purple’ and ‘Tianzi2hao’.

### The USAGT activity of different carrots *in vitro*

**Fig 8:**
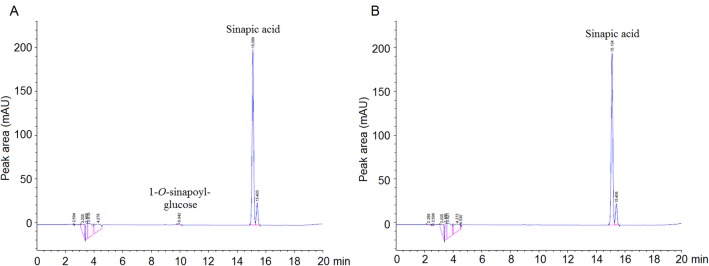
Chromatogram of USAGT activity *in vitro* by HPLC. (A) Reaction of crude protein from ‘deep purple’ carrot taproots. (B) Reaction of crude protein from non-purple ‘Kurodagosun’ carrot taproots.

As illustrated in the chromatogram, the retention time of the produced 1-*O*-sinapoylglucose and sinapic acid was 9.9 and 15.1 min, respectively (Fig 8A). It indicated that the crude protein extracted from the taproots of the purple carrots exhibited USAGT activity. However, no product was detected in Fig 8B, which indicated that the crude protein from the non-purple carrots did not exhibit USAGT activity. In total, USAGT activity was only detectable in the taproots of the purple carrots.

## Discussion

During the secondary metabolism of plants, numerous glycosides have already been isolated as biologically active compounds, and some of them have been widely used as important medicines [21]. Glycosyltransferases usually act in the final stages of plant secondary metabolism. They are used to stabilize and dissolve various low molecular mass compounds, such as flower pigments [22], and to regulate the action of functional compounds, such as plant hormones [23].

Purple carrot cultivars contain abundant anthocyanins in the taproots. In Glӓßgen’s report, the main activity for UDP-glucose glucosyltransferase in anthocyanin production used sinapic acid as substrate [24]. Sinapic acid plays an important role in maintaining the stability of cyanidin-based anthocyanins [8]. USAGT can transfer sinapic acid from UDP-glucose to the product of 1-*O*-sinapoylglucose [25]. 1-*O*-sinapoylglucose can serve as an acyl donor in acylation of anthocyanins and generate cyanidin 3-xylosyl (sinapoylglucosyl) galactoside in purple carrots. This final product helps stabilize the accumulation of anthocyanins [8].

Structure prediction of DcUSAGT1 is based on the model UGT72B1. UGT72B1 is the first UDP-glc-dependent NGT ever reported with glycosyltransferase-B topology [26]. The glycosyltransferase fold contains two β/α/β Rossmann domains, each corresponding to N- and C-terminals [27]. The structural model of DcUSAGT1 from purple carrot is similar to that of UGT72B1 from *A*. *thaliana*, which contains a similar motif. In purple carrot, sinapic acid may combine with the N-terminal domain, whereas UDP-glucose mainly binds to the C-terminal domain. The similar structural model between DcUSAGT1 and UGT72B1 indicated that DcUSAGT1 may also perform the function of UDP-glucose glycosyltransferase.

The Mr of glycosyltransferases is generally between 45 kDa and 60 kDa [28]. In this study, the expression vector hosting *DcUSAGT1* gene was constructed, then rDcUSAGT1 protein was purified to detect enzyme activity. Here, we focused on the use of sinapic acid as substrate for the determination of rDcUSAGT1 activity. Other studies also used other phenylpropanoids as substrates to detect enzymatic activity, such as cinnamic acid, caffeic acid, ferulic acid, and ρ-coumaric acid [11, 24]. The Mr of DcUSAGT1 was calculated to be 54.55 kDa. In a previous study, the gene encoding UDP-glucose: sinapic acid glucosyltransferase cloned from *B*. *napus* was predicted to have a Mr of 55.97 kDa [17]. Halaweish and Dougall found that crude extracts from wild carrots grown as cell cultures can catalyze the formation of 1-*O*-sinapoylglucose from sinapic acid and UDP-glucose. The activity of the purified enzyme was measured to identify its optimal temperature and pH value [15]. This report showed no significant difference in the amount of product formed at 30°C or 37°C, and optimum pH was between 6.5 and 7.0 [15]. In the present study, the peak of rDcUSAGT1 activity was at 30°C at pH value of 7.0. The *K*_m_ value of rDcUSAGT1 for 5 mM of sinapic acid was 0.59 mM with 50 mM of UDP-glucose under optimal conditions. The *K*_m_ value of rDcUSAGT1 was higher than that of UGT84A1 and UGT84A2 but lower than that of UGT84A3 for 1 mM of sinapic acid with 5 mM of UDP-glucose under optimal conditions in *A*. *thaliana* [10]. It indicated that the affinity of rDcUSAGT1 toward the substrate was high.

A previous study reported that genes *PAL3/PAL4*, *CA4H1*, *4CL1*, *CHS1*, *CHI1*, *F3H1*, *F3’H1*, *DFR1*, and *LDOX1/LDOX2* are powerful indicators for the production of anthocyanins in the taproots of purple carrot cultivars [29]. The transcripts for genes *CHS1*, *DFR1*, *F3H*, *LDOX2*, and *PAL3* accumulated at high levels in purple carrots, less in purple-orange carrots, and low or no transcripts were detected in orange carrots [30]. In the present study, significant differences in *DcUSAGT1* gene expression levels in purple carrots and non-purple carrots were detected. Significantly higher expression levels of the *DcUSAGT1* gene in the taproots of all three purple carrots than non-purple carrot taproots were detected, which is similar to previous expression patterns [29, 30]. It indicated that the low expression of *DcUSAGT1* gene in non-purple carrot taproots may be the determining step lost in anthocyanin production. To further prove the function of USAGT in carrot taproots, The USAGT activity of different carrot cultivars ‘Deep purple’ and ‘Kurodagosun’ was tested. The result showed that USAGT activity was detectable in the taproots of purple carrot *in vitro* instead of in non-purple carrot taproots.

In conclusion, the results presented here suggested that DcUSAGT1 is an efficient enzyme in the transformation of sinapic acid as substrate to product 1-*O*-sinapoylglucose and UDP. DcUSAGT1 maybe plays important roles in anthocyanins biosynthesis of purple carrot taproots. The study will provide a foundation for future understanding and manipulation of sinapic acid metabolism and anthocyanins synthesis in purple carrot taproots.

## Supporting Information

S1 File Relevant data underlying the findings described in the manuscript. Figure A. Nucleotide acid and deduced amino acid sequences of *DcUSAGT1* from purple carrot.
(DOC)

## Abbreviations

BSA: Bovine serum albumin
DTT: DL-Dithiothreitol
EDTA: Ethylenediaminetetraaceticacid
HPLC: High performance liquid chromatography
IPTG: Isopropyl β-D-1-thiogalactopyranoside
Mr: Relative molecular mass
ORF: Opening reading frame
PCR: Polymerase chain reaction
qRT-PCR: Quantitative real-time polymerase chain reaction
rDcUSAGT: Recombinant DcUSAGT
USAGT: UDP-glucose: sinapic acid glucosyltransferase
