# Supplementary material for: Identification and Characterization of DcUSAGT1, a UDP-Glucose: Sinapic Acid Glucosyltransferase from Purple Carrot Taproots
Source: PLoS One. 2016 May 12;11(5):e0154938. doi: 10.1371/journal.pone.0154938 (PMC4865115; doi:10.1371/journal.pone.0154938)
Supplement: S1 File — Figure A. Nucleotide acid and deduced amino acid sequences of DcUSAGT1 from purple carrot. (DOC) [file pone.0154938.s001.doc]

**Supplementary Information**

**Fig. A** Nucleotide acid and deduced amino acid sequences of *DcUSAGT1* from purple carrot

ATGCGGGGTTCTCATCATCATCATCATCATGGTATGGCTAGCGAATTGGCCCTTATGAGCCCTGTATATCAAGAC

1 M R G S H H H H H H G M A S E L A L M S P V Y Q D

TCCAAAAAGGATCTTGTTCATATCTTACTTGTGTCTTTCATAGGACAAGGCCATGTCAACCCTTTACTCAGACTA

26 S K K D L V H I L L V S F I G Q G H V N P L L R L

GGAAACCTCCTTGCTTCATCAGGTTTCCTTGTCACCTTCTCTTCCTGTTCGGAAGTCGGAAATTCCATGCGCAAG

51 G N L L A S S G F L V T F S S C S E V G N S M R K

GCTAACAACAACATCGATGAACTCGTTCCAGTTGGCGATGGGATGATCAGGTTTGAGTTTTTTGATGATGGATTA

76 A N N N I D E L V P V G D G M I R F E F F D D G L

CCCGAGAGTGATCCACGACGCCATGATCTCGACTTCTACATGCCTCACCTTGAGTTGCATGGTAAAGAGGCAGTC

101 P E S D P R R H D L D F Y M P H L E L H G K E A V

ACTGGCATTGTCAAGAAACACGAAAAAGAAGGGCGTCCGGTGTCATGCATCATCAACAATCCTTTCATTCCATGG

126 T G I V K K H E K E G R P V S C I I N N P F I P W

GTTTCTGACATCGCGGAGGCCTTAAGCATTCGTAATGCAGTGCTTTGGGTACAGTCCTGTGCTTGTTTTTCAGCT

151 V S D I A E A L S I R N A V L W V Q S C A C F S A

TATTACCATTACCACAATAAGTTGTCACAGTTTCCCTCCGAATCTGAACCCGAAATCGATGTCCAACTGCCATCT

176 Y Y H Y H N K L S Q F P S E S E P E I D V Q L P S

ATGCCTCTGTTAAAACATGATGAAATCCCAAGTTTCTTACACCCTTCAACTCCTTATCCAGCCTTAAGAAGAACA

201 M P L L K H D E I P S F L H P S T P Y P A L R R T

ATTCTAGCCCAATTCAAGAACTTGTCAAAACCATTCTGTGTACTAGTAGAAACATTTCAAGAACTCGAAAGTGAA

226 I L A Q F K N L S K P F C V L V E T F Q E L E S E

GTCATTGATTACATGTCCAAACTCTGCCCCATCAAGGCCATCGGCCCTCTATTCAAGAACCCGAAATCTCAACTT

251 V I D Y M S K L C P I K A I G P L F K N P K S Q L

TCTAACATCCAAGGCGATTGCTTGAAGCCCGTCGATGACTGCATCGATTTCCTGAACTCCAAAAGCCCCTCATCC

276 S N I Q G D C L K P V D D C I D F L N S K S P S S

GTTGTCTACATCTCTTTCGGCAGTGTTATCTCAATGAACCAAAAACAAACAAATGAACTTGCTCAAGGTCTTTTA

301 V V Y I S F G S V I S M N Q K Q T N E L A Q G L L

AACTCCGGAGTTTCTTTTCTCTGGGTTTTTAGGCCACCCCTTCCAGGTTTCGACGTAGCTGTACTACCCGAAAAA

326 N S G V S F L W V F R P P L P G F D V A V L P E K

TTCCTGGAAGCAGCTGGTGACAAGGGAAAAGTAGTGCAGTGGTGTTCACAGAAACAAGTCCTGGCAAGTCCTGCA

351 F L E A A G D K G K V V Q W C S Q K Q V L A S P A

GTGGCTTGTTTTCTGACACATTGCGGATGGAATTCGACGTTAGAGGCCTTAACAACTGGTGTCCCTGTGATAACC

376 V A C F L T H C G W N S T L E A L T T G V P V I T

TACCCTGCATGGGGTGATCAAGTCACTAATGCTAAGTTTTTAGTTGATGTGTTAAAAGTGGGAGTCAGGTTAAGT

401 Y P A W G D Q V T N A K F L V D V L K V G V R L S

AGAGGGAACCAATCTAAAAAAAATGTCATTTCCAGGGACGATATCGAAAAATCTTTAAGAGAAGCTACCATTGGG

426 R G N Q S K K N V I S R D D I E K S L R E A T I G

GAAAATGCAGCAGAGATTAAAAGGAATGCATTGAAGTGGAAGGAGGCGGCAGAGGACGCGGTGGCAGAGGGCGGT

451 E N A A E I K R N A L K W K E A A E D A V A E G G

TCTTCTGATCGGAACTTGAAAGAGTTTGTGGATAAGCTGATGATGAACTGA

476 S S D R N L K E F V D K L M M N *
